# Comparison of Quantitative Real-Time PCR and Digital PCR to Detect the Polyomavirus in Merkel Cell Carcinoma

**DOI:** 10.3390/v14102195

**Published:** 2022-10-05

**Authors:** Martina Barchitta, Andrea Maugeri, Elisabetta Campisi, Roberta Magnano San Lio, Giuliana Favara, Hector Jose Soto Parra, Lucia Salvatorelli, Gaetano Magro, Guido Basile, Antonella Agodi

**Affiliations:** 1 Department of Medical and Surgical Sciences and Advanced Technologies “GF Ingrassia”, University of Catania, Via S. Sofia 87, 95123 Catania, Italy; 2 Medical Oncology, Azienda Ospedaliero Universitaria Policlinico “G. Rodolico-S. Marco”, 95123 Catania, Italy; 3 Department of General Surgery and Medical-Surgical Specialties, University of Catania, Via S. Sofia 78, 95123 Catania, Italy

**Keywords:** Merkel cell polyomavirus, Merkel cell carcinoma, FFPE skin samples, real-time qPCR, digital PCR

## Abstract

Merkel cell polyomavirus (MCPyV) prevalence in Merkel cell carcinoma (MCC) cases is controversial. The detection and quantification of MCPyV DNA is mainly performed by PCR techniques using formalin-fixed, paraffin-embedded (FFPE) tissues. The aim of this study is to compare the performance of two different molecular techniques, specifically the quantitative Real-Time PCR (qPCR) and digital PCR (dPCR). Samples from 31 cases of MCC excisional surgical biopsies were analyzed. DNA extraction and purification from clinical samples were performed using the QIAcube Qiagen automated nucleic acid extractor. After the extraction, MCPyV was detected by qPCR and dPCR using specially designed primers and probes. Of the 31 MCC samples under study, the MCPyV genome was detected in 11 samples (35%) by qPCR compared with 20 samples (65%) detected by dPCR. Notably, 65% of primary tumors were positive for MCPyV (15/23). The viral genome was detected in 75% of tumors located at UV-exposed sites (6/8), 55% of tumors at partially UV-protected sites (5/9), and 67% of tumors at UV-protected sites (4/6). Our results showed a better sensitivity of dPCR in detecting the MCPyV genome in MCC samples compared with traditional qPCR techniques.

## 1. Introduction

Merkel cell carcinoma (MCC), also known as neuroendocrine carcinoma of the skin, is a rare and extremely aggressive tumor with a propensity for sun-damaged skin. The commonest sites affected are the head and neck (50%) (mainly the eyelid and periorbital region) and the extremity (40%). A primary tumor of the trunk (10%) and only occasional cases are reported on sun-protected sites such as the ear canal, oral and nasal mucosa, vulva, or penis. Caucasians have an estimated annual incidence of 0.23 per 100,000, while people with black skin have an estimated incidence of 0.01 per 100,000. Most patients affected by MCC are in their seventh decade or elder, while only 5% are under the age of 50. MCC affects children only exceptionally. Merkel cell carcinoma can also arise in immunocompromised patients such as after solid organ transplantation [1].

MCC usually presents as a painless, rapidly growing, single, red, or purple cutaneous nodule or sometimes as an indurated plaque that will elude diagnosis until histological examination is performed [2]. MCC is characterized by regional nodal and/or distant metastases, a high incidence of local recurrence, and a high mortality rate [2]. MCC has a mortality of almost 33% at 3 years, which is higher than melanoma (15%). The Surveillance, Epidemiology, and End Result (SEER) reported a three-fold increase in MCC from 0.15 to 0.44 per 100,000 annually (from the years 1986 to 2001) [3]. This trend is ongoing, and several factors likely contribute to this. An aging population, an increased total sun exposure and a higher number of immunosuppressed individuals are the most important factors involved. Moreover, the advent of the immunohistochemical markers, especially neuroendocrine markers (chromogranin A and synapthophisin) as well as cytokeratin-20, improved recognition of this disease. Merkel cell carcinoma (MCC) can be classified on the basis of the association with Merkel cell polyomavirus (MCPyV), obtaining two groups with different tumorigenesis pathways [4]. However, MCPyV prevalence in MCC cases is controversial, probably due to differences in the management and molecular analysis of biological samples.

MCPyV is a non-enveloped, small, circular, double-stranded DNA virus that integrates into the tumor genome [5]. MCPyV is part of the human skin microbiome, and it has been detected in up to 80% of MCC samples, as well as in other non-melanoma skin tumors. The virus determines asymptomatic infections of the skin, and it is highly prevalent in the population. There is a growing body of evidence to support a critical role in the pathogenesis of MCC, including the identification of viral sequences integrated in the same site within primary tumors and their metastases [4,6,7]. MCPyV-related tumorigenesis follows a model of multi-step progression, in which a sequence of different events is required to induce the neoplastic transformation. Primarily, the MCPyV genome is linearized and integrated into the host genome after a concurrent DNA-damaging event, such as UV exposure. Second, infected cells are forced to express two viral oncoproteins: small tumor antigen (sT) and large tumor antigen (LT). While sT has intrinsically oncogenic activity, by inhibiting the proteasomal degradation of cyclin E and c-Myc, LT acquires pro-tumorigenic activity only when mutations of the 3′ end of the gene lead to the loss of the protein C-terminus. Indeed, the truncated LT inactivates the tumor suppressor Rb, driving uncontrolled cell proliferation [6]. Following tumor formation, numerous mechanisms contribute to tumor cell survival in the presence of a destructive immune response. In addition, MCPyV-specific T cell responses are detected both locally and systemically in patients with MCC, but the frequent expression of PD-L1 by cancer cells inactivates their effects by inducing T cell exhaustion. In this context, the defective expression of HLA class-I by tumor cells may hamper antigen presentation, further promoting immune evasion [6].

According to the fact that MCC most commonly affects sun-exposed areas of the skin, MCC prevalence is higher in Caucasians as well as in regions with elevated UV radiation [3,7]. Recently, several studies showed that MCPyV-negative MCCs have a very high mutation burden associated with UV exposure. These mutations are predominantly C > T transitions, characteristic of UV-induced DNA damage [8,9]. Virus-negative MCCs have a considerably higher mutational burden as compared with virus-positive tumors, supporting that they have a different pattern of genomic alterations [7]. MCPyV-negative MCCs harbor recurrent, clonal mutations inactivating RB1, TP53, and other genes that are implicated in the Notch signaling [6].

Several studies suggested a primary role for UV light in MCPyV-negative MCCs’ mutagenesis but not in MCPyV-positive ones. Contrariwise, in MCPyV-positive tumors, UV light could promote rare mutations required for MCPyV integration. and UV radiation could modify the tumor microenvironment such as driving immunosuppressive effects [8]. In several studies, formalin-fixed, paraffin-embedded (FFPE) tissue samples were analyzed by quantitative Real-Time PCR (qPCR) [10,11,12]. More recently, some studies introduced digital PCR (dPCR) as a method to detect and quantify the virus [13]. Since MCPyV prevalence in MCC cases is controversial, probably due to differences in the management and molecular analysis of biological samples, we aim to evaluate the different performance of qPCR and dPCR for the detection and quantification of MCPyV in FFPE samples from patients with MCC.

According to the association with MCPyV, MCCs can be split in two distinct groups characterized by different tumorigenesis steps. However, it is not known if virus-positive and virus-negative tumors originate from the same cell precursor. Both tumorigenesis pathways lead to RB and p53 inactivation, even if the transformation is associated with two different causes (virus and UV-induced mutations). Immunogenicity of both carcinoma groups is high due to the presentation of neoantigens or viral peptides [14]. Despite the association between MCPyV and MCC development, only limited information is available about the association of viral factors with tumor development. In this context, MCPyV viral load is supposed to correlate with clinical disease severity or progression, but results are controversial [15]. In this scenario, the primary purpose of this study is to detect MCPyV presence in skin lesions’ biopsies from patients with a histologically/immunohistochemically proven diagnosis of MCC. In particular, we compare two different molecular techniques, namely the qPCR and dPCR. The secondary aim is to investigate differences between patients with lesions in sun-protected or sun-partially protect sites and those with sun-exposed sites’ lesions, analyzing the possible association with viral detection and tumor localization.

## 2. Materials and Methods

**Figure 1:**
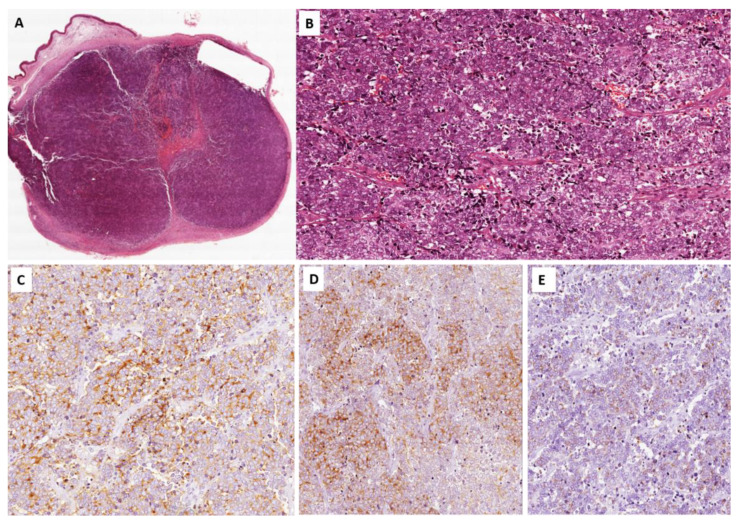
Merkel cell carcinoma. (**A**) Histological examination showing a tumor nodule involving the dermis. (**B**) Higher magnification showing a malignant tumor composed of small- to medium-sized round cells with vesicular nuclei showing “salt and pepper” appearance. Neoplastic cells showing cytoplasmic immunoreactivity for chromogranin A (**C**), synaptophysin (**D**), and cytokeratin 20 (**E**).

We collected data corresponding to samples of 31 MCC skin tumors, which were biopsied or surgically resected between 2001 and 2022. Pathological reports and all available slides and paraffin blocks were retrieved from the archives of Anatomic Pathology, Department G.F. Ingrassia, University of Catania. At the time of diagnosis, tumor samples were fixed with formalin and then embedded in paraffin. To confirm MCC diagnosis, immunostaining of Cytokeratine20 (CK20) and one or more neuroendocrine markers (chromogranin A, synaptophysin, or neuron-specific enolase) was used (Figure 1).

To isolate DNA from the formalin-fixed paraffin-embedded samples, 10-micron thick sections (five from each sample) were treated with 1 mL of chloroform, then vortexed for 10 min, and finally incubated for 1 h at room temperature. Centrifugation (12,000 rpm for 5 min) was used to precipitate out cell pellets which were dried and resuspended in 1 mL of pure ethanol and then vortexed for 5 min. The next step was a second centrifugation (12,000 rpm for 5 min) followed by ethanol elimination. Finally, samples were resuspended in 200 µL of water, after which the genome was extracted using the QIAcube Qiagen automatic Nucleic Acid Extractor.

After the extraction, MCPyV was first detected using qPCR. Briefly, the reaction contains 11 µL of a 2× PCR super mix (EvaGreen, Biotium, Fremont, CA, USA), 0.4 µL of each working primer solution (final concentration ranging between 0.18 µM and 0.20 µM each), and 10.2 µL of DNA (~100 ng per reaction). The cycling conditions were as follows: 10 min at 95 °C, 35 cycles of: 30 s at 94 °C, 1 min at 57 °C, and 10 min at 98 °C. Similarly, all samples were analyzed using the dPCR. The reaction was performed in a final volume of 20 μL, containing 13.3 µL of super mix (EvaGreen), 4 µL of primers (2 µL of forward and 2 µL of reverse), and 22.7 µL of DNA. The cycling conditions were as follows: 10 min at 95 °C, 35 cycles of: 30 s at 94 °C, 1 min at 57 °C, and 10 min at 98 °C. In both cases, primers used are as follows: RQMCPyV_LT_1F 5′-CCACAGCCAGAGCTCTTCCT-3′ and RQMCPyV_LT_1R 5′-TGGTGGTCTCCTCTCTGCTACTG-3′.

## 3. Results

The median age of the patients was 74 years, with a range from 49 to 92 years. A total of 41% of patients were female (12/29) with a median age of 74 years, while the remaining 59% were male (17/29) with a median age of 64 years. Two patients had a recurrence of disease: for them, two biopsies were analyzed distinctly. Overall, 16 biopsies presented positive margins of resection. Four biopsies were lymph node/metastasis, 23 were primary tumors, while, for four tumors, localization was not reported. All tumors included in the present study exhibited the classic morphology of Merkel cell carcinoma and expressed diffusely chromogranin A, synaptophysin, and cytokeratin 20 (dot-like expression pattern).

**Figure 2:**
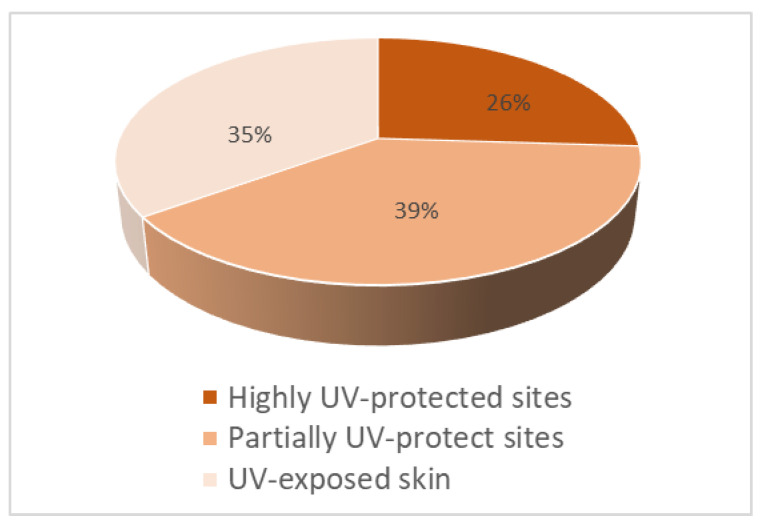
Distribution of primary lesions by their UV exposure.

MCPyV sequences were detected in 11 MCC samples (35%) through qPCR and in 20 MCC samples (65% of cases) through dPCR, respectively. Thus, dPCR showed higher sensitivity than did qPCR in FFPE samples. In fact, 9 of 31 (29%) biopsies were positive by dPCR and negative by qPCR. The 65% of primary tumors (15/23) were MCPyV-positive lesions, and the MCPyV genome was also detected in one lymph node metastasis (1/3). Although sun exposure is strongly associated with MCC, as in melanoma, MCC can arise in the absence of significant UV exposure. The anatomic distribution of the 26 tumors seen in our study further supports this theory. In our series, 26% (6/23) of biopsies presented on partially UV-protected sites (lower limbs), 39% (9/23) of patients had tumors on highly sun-protected sites (trunk, back, and buttock), while only 35% (8/23) of cases presented on UV-exposed skin (head and upper limbs). As seen in our series, the localization of 65% of lesions was in sun-protected and partially sun-protected sites, while only the 35% cases presented in sun-exposed sites (Figure 2). MCPyV was detected in 75% of UV-exposed site tumors (6/8). In all five head MCC biopsies, four of them resulted MCPyV positive samples, while 2/3 upper limbs’ samples were MCPyV-positive tumors. MCPyV was detected in 55% of partially UV-protected site tumors (5/9), and 66.6% of UV-protected sites were MCPyV-positive tumors (2/3). These results confirmed that MCC can arise in the absence of significant UV exposure, and MCPyV is also involved in UV-exposed sites’ tumorigenesis (Table 1).

## 4. Discussion

Since formalin-fixed and paraffin-embedded tissues contain a high degree of tissue damage, reducing the amount of usable DNA molecules for downstream studies, the quality of the detection assay is important when FFPE tissues are analyzed. Paik et al. reported that head and neck MCCs show a lower frequency detection of MCPyV compared to other sites, and they also reported that in a cohort of Australians, MCPyV was present in only 18% of cases [16]. PCR techniques are used with FFPE tissues, and DNA could be damaged during the processing of the specimens. This impaired sensitivity of the assay (fresh or FFPE) could explain some of the differences in virus detection [17]. Two studies showed that MCPyV detection rates were lower in FFPE tissues than in frozen samples [5,18]. A recent study compared viral load level in both types of samples (FFPE tissue and frozen specimens) from the same MCC patient [19]. High viral loads detected in frozen tissue samples were higher than in corresponding FFPE tissues, confirming that fresh-frozen tissues are undeniably the best type of samples for PCR assays because of the limited degradation of DNA compared with FFPE ones [20]. Frozen tissues are rarely available, and hence—in their absence—FFPE tissues are great and reliable surrogates. These data underscore the need for accurate quantification methods. Since the viral load in this kind of sample is usually low, the real prevalence of viral DNA could be underestimated [21]. At present, the detection and quantification of MCPyV DNA in tissue specimens is mainly performed by qPCR [10,11,12]. Recently, dPCR was introduced to detect the viral load in clinical samples. Urso and colleagues, using the dPCR assay, found a significantly higher prevalence of MCPyV DNA in FFPE biopsies than in skin from healthy subjects [21]. Arvia et al. evaluated the performance of both qPCR and dPCR assays for MCPyV detection and quantification in FFPE tissue samples. Regarding reproducibility and repeatability, the two approaches resulted in equivalent outcomes, without a significant difference between viral loads measured by the two methods. However, dPCR was able to detect MCPyV in a higher number of specimens, so it can be considered a better method to detect MCPyV in FFPE cutaneous samples, mostly these containing a low copy number of viral DNA [13]. In our study, dPCR had better sensitivity in detecting the MCPyV genome in MCC samples when compared with the traditional qPCR technique. However, some limitations should be considered when interpreting our findings. The limited sample size and the absence of further information on the gold standard diagnostics of each case did not allow us to evaluate factors that might affect the sensitivity of qPCR and dPCR. It is also worth underlining that we did not aim to validate qPCR and/or dPCR for detecting the MCPyV genome in MCC samples. However, our intention was to report differences—in terms of sensitivity—between these techniques. For this reason, we did not include a control group of negative skin biopsies, which represents a weakness of our study. Another limitation consisted of the absence of an alternative assay with a second primer pair that could have improved the sensitivity of the analysis. With these considerations in mind, validation studies should be encouraged to evaluate the analytical sensitivity of dPCR in detecting the MCPyV genome in MCC samples. These studies should include a higher number of samples to evaluate the influence of other factors (e.g., gender, tumor site, tumor stage, etc.) on the observed analytical differences.

In conclusion, dPCR can detect MCPyV DNA in a higher number of specimens and can be considered a better method to analyze FFPE cutaneous samples. An improvement in MCPyV detection is crucial for further studies to evaluate the potential association between viral infection, clinical features, patient age and sex, disease progression, and survival.

## Figures and Tables

**Table 1:** Characteristics of the study population.

Patient	Age	Sex	Biopsy Anatomical Region	Margins Involved	MCPyV
1	N.A.	Female	Cerebellopontine Angle	No	No
2	80	Female	Right Forearm	No	No
3	N.A.	Female	Left Leg	No	Yes
4	86	Female	Left Leg	No	Yes
5	62	Male	Left Leg	Yes	No
6	76	Female	Right Thigh	Yes	No
7	85	Male	Left Gluteus	Yes	Yes
8	75	Female	Left Cheekbone	Yes	Yes
9	63	Female	Left Leg	Yes	No
10	75	Male	Left Hand	Yes	Yes
11	80	Male	Left Gluteus	Yes	Yes
12	80	Male	Right Knee	Yes	No
13	60	Female	Left Gluteus	No	Yes
14	61	Female	Lymph Node of The Left Groin	No	No
15	N.A.	Male	Cheek	Yes	No
16	64	Male	Back	No	Yes
17	67	Male	Lymph Node of The Left Groin	No	No
18	88	Male	Inferior Lip	Yes	Yes
19	88	Male	Chin	Yes	Yes
20	49	Male	Left Thigh	Yes	Yes
21	87	Male	Chest	No	No
22	92	Female	Right Cheek	Yes	Yes
23	55	Female	Right Tigh	No	Yes
24	56	Male	Left Leg	Yes	Yes
25	77	Female	Gluteus	No	Yes
26	76	Male	Lymph Node of The Armpit	Yes	No
27	81	Female	Leg	No	Yes
28	83	Male	N.A.	Yes	Yes
29	N.A.	N.A.	N.A.	N.A.	Yes
30	78	Female	N.A.	N.A.	Yes
31	N.A.	N.A.	N.A.	N.A.	Yes

Abbreviations: N.A., not available data.

## Data Availability

Data available on request from the corresponding author.
